# The complete mitochondrial genome of *Sarcocheilichthys davidi* and its phylogeny

**DOI:** 10.1080/23802359.2018.1456980

**Published:** 2018-04-01

**Authors:** Tian Wu, Tao Zhang, Yuanchao Zou, Mei Chen, Qing Deng

**Affiliations:** Conservation and Utilization of Fishes Resources in the Upper Reaches of the Yangtze River Key Laboratory of Sichuan Province, College of Life Sciences, Neijiang Normal University, Neijiang, China

**Keywords:** *Sarcocheilichthys davidi*, mitochondrial genome, phylogenetic analyses

## Abstract

*Sarcocheilichthys davidi* is a small-size ornamental freshwater species. In the present study, the complete mitochondrial genome of *S. davidi* was first determined using the next-generation sequencing technology. The result showed that the circular mitogenome was 16,675 bp in length, contained 13 protein-coding genes (PCGs), two ribosomal RNA (rRNA) genes, 22 transfer RNA (tRNA) genes, and two non-coding regions: origin of light-strand replication (OL) and displacement loop locus (D-loop). In addition to ND6 and eight tRNA genes, most mitochondrial genes were encoded on the heavy strand. Furthermore, the overall base composition was 29.97% A, 26.12% T, 26.86% C, 17.04% G, respectively, and showed AT-rich feature (56.09%). Moreover, phylogenetic analysis based on the tandem 13 PCGs nucleotide sequences indicated that *S. davidi* had a close relationship with *S. snigripinnis* and *S. variegatus wakiyae*. The complete mitogenome sequence of *S. davidi* would contribute to the better understanding of molecular systematics, selection, conservation, species identification and evolution of this species.

*Sarcocheilichthys davidi*, belonging to the Gobioninae subfamily of the Cyprinidae family, is a small-size ornamental freshwater species. It is distributed in the tributary of the upper reaches of the Yangtze River. This fish is an important economic fish, but study on mitochondrial gene sequencing of the fish has not been reported. In this study, the complete mitochondrial DNA sequence was first determined by the next generation sequencing (NGS).

The specimens were obtained from Neijiang, Sichuan Province of China (29°35′22.35″N, 105°03′52.22″E) in September 2017, and were stored in Zoological Specimen Museum of Neijiang Normal University (accession number: 20170921BB01). A 30–40 mg fn clip was collected and preserved in 95% ethanol at 4 °C. Total genomic DNA was extracted with a Tissue DNA Kit (OMEGA E.Z.N.A., Norcross, GA) following the manufacturer’s protocol. Subsequently, the genomic DNA was sequenced using the NGS, and then the mitogenome was assembled using *S. sinensis* as reference.

The complete mitogenome of *S. davidi* was determined to be 16,675 bp in size (GenBank Accession number MG797641). It is composed of 13 protein-coding genes (PCGs), 22 transfer RNA (tRNA) genes, two ribosomal RNA (rRNA) genes, and two non-coding regions: origin of light-strand replication (OL) and displacement loop locus (D-loop). The total nucleotides composition of the *S. davidi* mtDNA was 29.97% A, 26.12% C, 26.86% T, 17.04% G, with 56.09% A + T, which was basically consistent with those of other teleost species (Chen et al. 2015; Li et al. 2015; Jia et al. 2016). Among 37 genes, nine genes (ND6 gene and eight tRNA genes) were encoded on the L-strand, and the remaining 28 genes were encoded on the H-strand. In 13 PCGs, the shortest one was ATP8 (165 bp in length), whereas the longest one was ND5 (1833 bp in length). COI initiated with a GTG while all other PCGs started with an ATG, which was similar to *S. sinensis.* Moreover, seven PCGs terminated with TAA codon, except three genes (COII, COIII, Cyt b) ended with incomplete codon (T–) and three genes (ND1, ND4, ND6) used TAG as stop codon. Which was a bit different from *S. sinensis* and *S. nigripinnis* (ND2, ND3, and ND4 of the two fishes stopped with TA-codon) (Li et al. 2014; Wang et al. 2016). Furthermore, 22 tRNA staggered in two rRNAs and 13 PCGs, ranging from 68 bp (tRNA^Cys^) to 76 bp (tRNA^Leu^, tRNA^Lys^) in length. Additionally, the 12S rRNA gene (959 bp) and 16S rRNA gene (1688 bp) were located between the rRNA^Phe^ and tRNA^Leu^ (UUR) genes and separated by the tRNA^Val^ gene. The D-loop region (1005 bp) was located between tRNA^Phe^ and tRNA^Pro^. Gene sequence analysis showed that there were 25 bp nucleotide overlaps. A total of six pairs overlap on the same type of genes, between 1 and 7 bp in length (tRNA^Ile^-tRNA^Gln^, tRNA^Thr^-tRNA^Pro^, ATP8-ATP6, ATP6-COIII, ND4L-ND4, ND5-ND6). It was also found that 14 genes intervals (59 bp) ranged in length from 1 to 30 bp and the longest (OL) was between tRNA^Asn^ and tRNA^Cys^.

Thus far, the mitochondrial genes have been widely used for inferring phylogenetic relationships (Boore et al. 2005). A phylogenetic tree was constructed based on the 13 PCGs nucleotide sequences of *S. davidi* and other Gobioninae fishes using the neighbour-joining (NJ) method and maximum likelihood (ML) method (Zou et al. 2017). In addition, *Channa argus* were defined as an outgroup species. The generated NJ tree and ML tree were summarized and the same topological structure tree was obtained. The analysis of two kinds of trees showed that Gobioninae subfamily was divided into two clades: *Sarcocheilichthy* and (*Squalidus, Hemibarbus*) (Figure 1). In addition, two topologies all showed *S. davidi* and sister group (*S. snigripinnis*, *S. variegatus wakiyae*) had a close relationship, which indicated that the rate of evolution of these three species was roughly equivalent.

**Figure 1.**
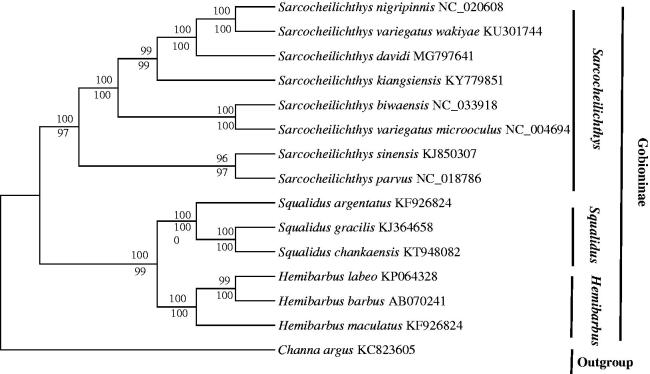
The phylogenetic analyses investigated using maximum likelihood (ML) and neighbour-joining (NJ) methods analyses indicated evolutionary relationships among 15 taxa based on the nucleotide alignments of 13 protein-coding genes. The tree topologies produced by ML and NJ analyses were equivalent. NJ posterior probabilities and ML bootstrap values are shown on the nodes. *Channa argus* (GenBank: KC 823605) was used as the outgroup.
